# Host-guest complexes of imazalil with cucurbit[8]uril and β-cyclodextrin and their effect on plant pathogenic fungi

**DOI:** 10.1038/s41598-018-21156-9

**Published:** 2018-02-12

**Authors:** Naji Al-Dubaili, Khaled El-Tarabily, Na’il Saleh

**Affiliations:** 1 Chemistry Department, College of Science, United Arab Emirates University, P.O. Box, 15551 Al-Ain, United Arab Emirates; 2 Biology Department, College of Science, United Arab Emirates University, P.O. Box, 15551 Al-Ain, United Arab Emirates

## Abstract

We report the control of imazalil (IMZ) antifungal activity utilizing its non-covalent assembly with β-cyclodextrins (β-CD) and cucurbit[8]uril (CB8) macrocycles, as well as its stimuli-responsive disassembly with cadaverine. The NMR results are consistent with inclusion of a single IMZ molecule inside the cavities of either CB8 from its aromatic site or β-CD from its aliphatic end. Efficient complex formation with both host molecules and controlled released upon the addition of cadaverine is supported by NMR measurements. The stimuli-responsiveness of the same host-guest assemblies with cadaverine was validated against seven economically important plant pathogenic fungi which cause agriculturally important plant diseases across the globe. While loading the drug into macrocycles cavities suppressed its activity, subsequent adding of cadaverine efficiently restored it up. The results in the present paper enable researchers working in the area of mycology and plant pathology to inhibit or reduce the fungal growth on demand in order to control these economically important plant pathogenic fungi.

## Introduction

Modern researches in supramolecular chemistry have geared towards the design and synthesis of new supramolecular architectures in water that could bridge the molecular gap between synthetic small molecules on the one hand and biomolecules such as proteins and cellular membranes on the other hand^1^. These synthetic supramolecular systems, which were initially inspired by biological systems, are based on non-covalent interactions of mostly different small organic molecules. The interactions include hydrogen-bonding, π−π stacking, electrostatic, van der Waals force, and hydrophobic/hydrophilic attraction^2^. The increased knowledge over science of chemistry, particularly intermolecular interactions and molecular recognitions, has enabled the generation of well-defined, unprecedented advanced materials that could simulate to a certain degree the complexity and functionality of biological systems that they were initially inspired from^2^. At least, gaining control over theses supramolecular interactions could lead to modulations of specific biological processes^3^. When compared to routine covalent modifications, the non-covalent method considered inexpensive and environmentally safe approach. On the one hand, it bypasses the burdens in multiple organic synthesis steps^4^, and on the other it may combine already existing substrates, whose safety and low-toxicity have already been confirmed^5,6^. Low toxicity, in particular, motivated more researchers to utilize supramolecular systems to modulate biological processes^3^.

Cyclodextrins (CDs)^5^, and Cucurbit[n]uril (CBs)^7^ are examples of hosts that bind to guests by noncovalent interactions. CDs consist of glucopyranose units with a hydrophobic central cavity^5^. There are three main types of CDs (α, β and γ), the difference between them is the number of glucopyranose units in their structures which is six, seven and eight, respectively^5^. CBs composed of glycoluril units connected by methylene groups^7,8^. While CBs have much higher affinity to bind guest molecules as high as 10^15^ M^−1 ^^9^, the binding constant (*K*) for CDs-based host−guest complexes can reach 10^4^ M^−1 ^^5^. Our group has become interested on specifically utilizing CBs hosts due to their distinct preferential binding to cationic guests. This important hosting property is due to the ion-dipole interactions between carbonyl portals and cationic guests, leading to an increase in their acid-dissociation constant^10^. However, the important advantage of CDs is their ability to increase the solubility of poorly soluble drugs^5^. Our group^10–12^ among others^13–16^ have utilized CBs-induced p*K*_a_ shifts to simulate natural receptors in their modulation of acid-base equilibrium^17^ that is displayed by some potential bioactive molecules^10,13^, pharmaceutical drugs^11,12,14,15^ and hormones^16^. Generally, supramolecular approach utilizing CBs and CDs has also been employed to control several biological phenomena such as enzymatic activities^18^, or chemical sensing in fish^19^, just to name a few. Moreover, recognitions by CBs and CDs for biomolecules^20^ have also been confirmed in several reports, such as vitamins, peptides, proteins, and amino acids.

Supramolecular chemistry through intermolecular interactions inherently endow the final composite with reversibility and responsiveness to external stimuli, such as pH, light, electrical signal, heat, chemical competitor, etc^21^. In this regard, self-assembly process describes transformation of small building blocks into one defined aggregate under certain conditions, while self-sorting process is simultaneous occurrence of multiple self-assemblies into multiple well-defined aggregates under similar conditions. If building blocks of those self-assembling or self-sorting systems re-configured in responding to certain stimuli, the systems said to be stimuli-responsive systems. There have been several host-guest systems reported in aqueous solution for developing biologically relevant molecules with stimuli-responsive abilities. For examples, cucurbit[6]uril (CB6)-encapsulated 1-methylcyclopropene (1-MCP), an ethylene antagonist, that responses to sodium bicarbonate and benzoic acid as chemical competitors^22^, host-guest complexes of auxin plant hormones with cucurbit[7]uril (CB7) that response to pH, in which only at low pH inclusion complexes were formed^16^, CB7-encapsulated cadaverine (CAD) that responses to photoinduced pH jump resulting from photoreaction of 2-nitrobenzaldehyde^23^, and phototriggered release of memantine from CB7 cavity utilizing reversibly switchable photochromic system^24^. Aside from studies in water media, the validations of reversible response of supramolecular host-guest nanostructured materials to external stimuli *in vitro*^25–28^ or even *in vivo*^29^ have also been confirmed over the past two years. Benefits of these studies varies from confirming cellular uptake of either free or CB-complexed guest to unfolding mechanism of cellular uptake^30^.

**Figure 1:**
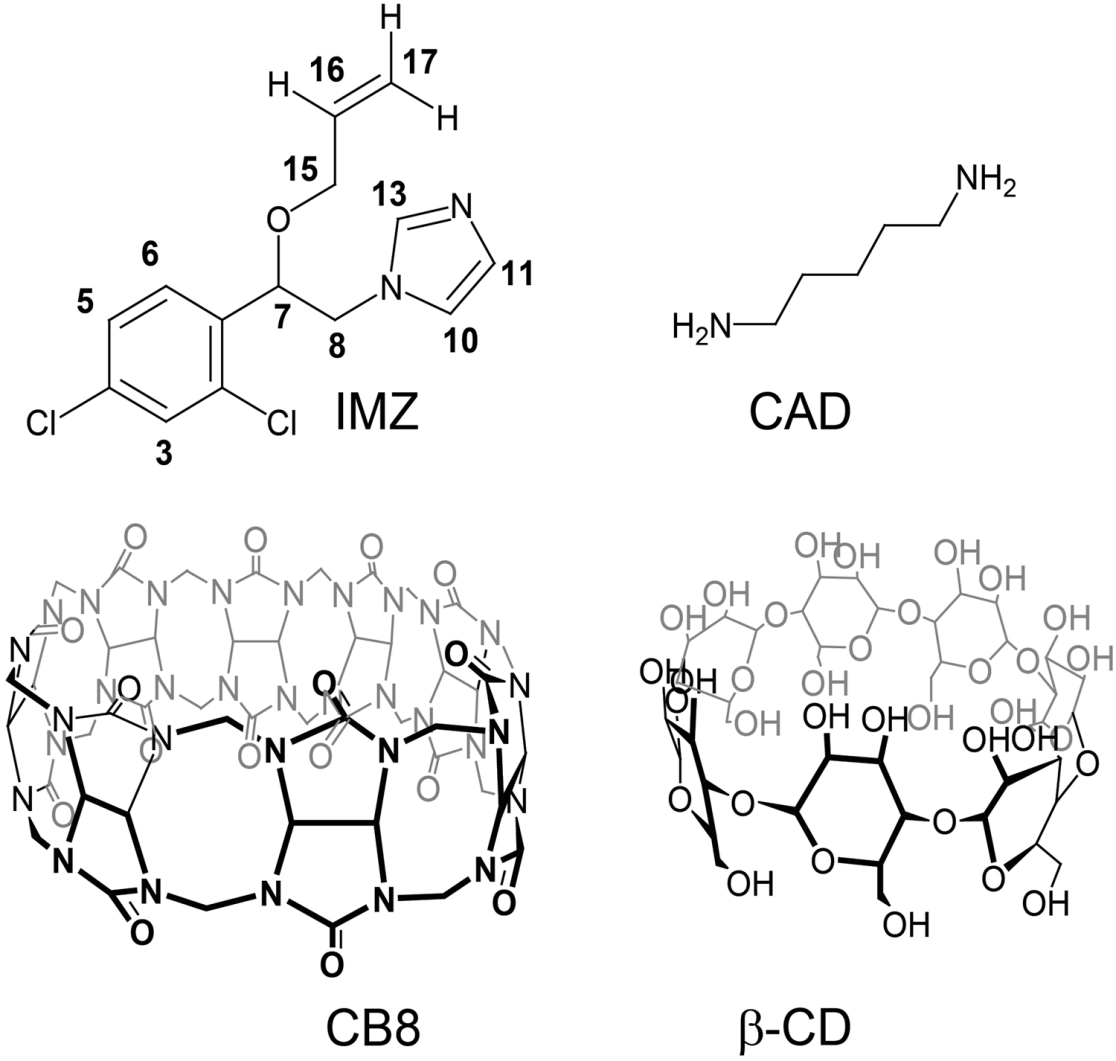
Chemical structures of model antifungal drug imazalil (IMZ), model chemical stimulus cadaverine (CAD), and model molecular containers cucurbit[8]uril (CB8) and β-cyclodextrin (β-CD).

In the present work, control over antifungal drug action against several economically important plant pathogenic fungi by supramolecular approach in response to CAD was demonstrated *in vitro* utilizing cucurbit[8]uril (CB8) and β-cyclodextrins (β-CD) as model macrocycles, as well as imazalil (IMZ) as a model antifungal drug (Fig. 1).

Even though, several articles have reported the encapsulation of biocides by CDs, CBs and other macrocycles (see discussion below), these papers did not address the potential use of macrocycles in controlling rate of microbial growth when the final composite is responding to one chemical stimulus as in the present study. The results of controlling fungal growth should attract attentions of many microbiologists, especially those who are working in the area of mycology and plant pathology to inhibit or reduce the fungal growth on demand in order to control these economically important plant pathogenic fungi. From a broader prospective, the work demonstrates the ability to manipulate on demand live biological systems utilizing classical organic molecules, which is a contemporary research in biomolecular sciences.

## Results

### Interactions of IMZ with β-CD and CB8

**Figure 2:**
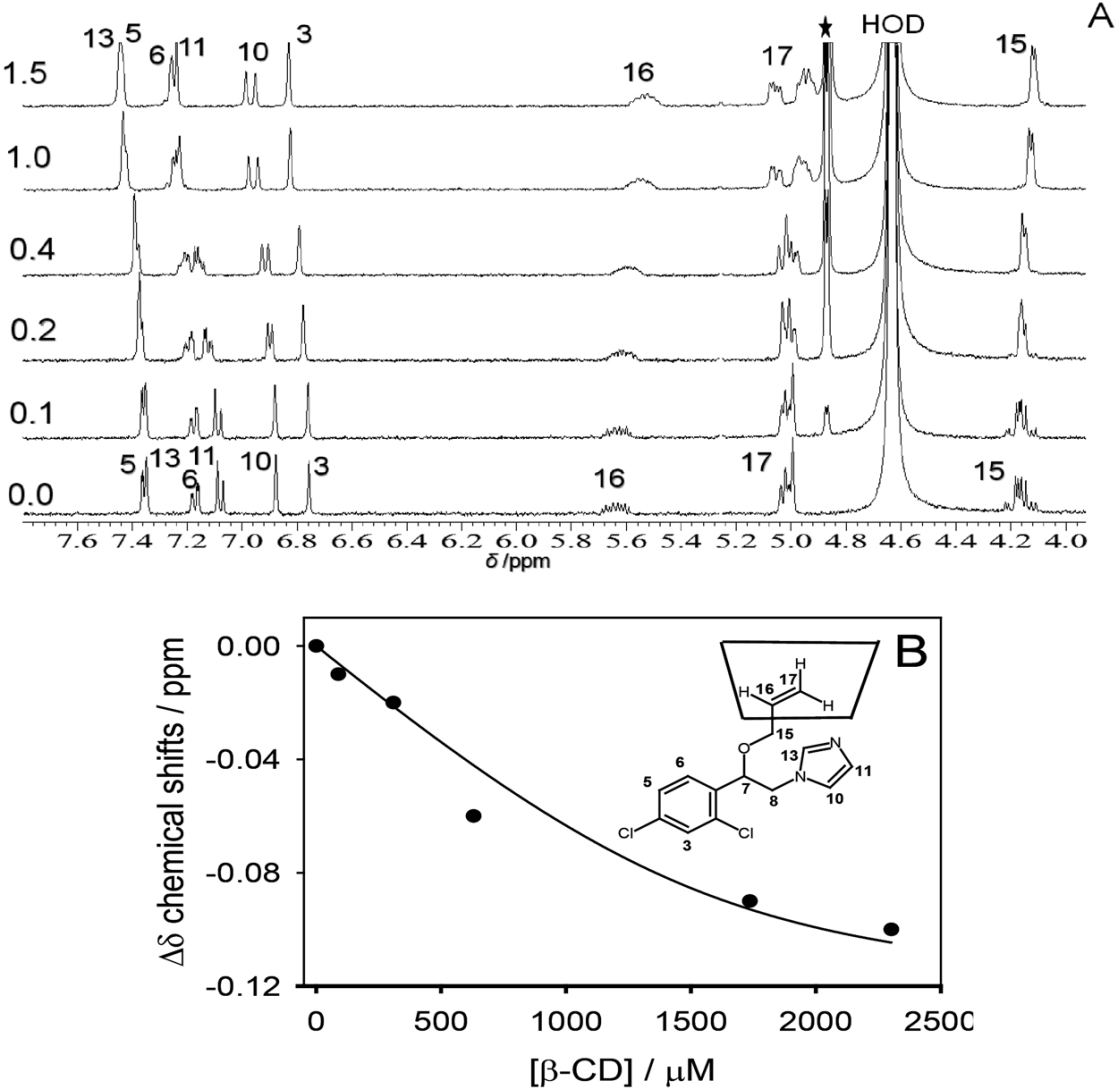
^1^H-NMR titration of IMZ with β-CD (0–1.5 equiv.) in D_2_O at pD 8.0 (400 MHz): (**A**) Spectral changes; (**B**) Nonlinear fitting plot of the chemical shift (ppm) at ~5.6 ppm versus concentration of β-CD in M. *K* was evaluated as (5.3 ± 0.9) × 10^3^ M^−1^. The inset in B shows a schematic representation of the resulted complex. HOD = Solvent Peak and *β-CD peaks.

The NMR titration experiment in D_2_O of neutral IMZ at pD 8.0 (see pH titration results; Figure S1 in the Supporting Information), in which the concentration of the drug was kept constant and different equivalents of β-CD, were subsequently added has resulted in the spectra illustrated in Fig. 2A.

The results confirmed moderate interactions of the drug with β-CD (binding constant ~5000 M^‒1^) and revealed the binding mode and stoichiometry inside β-CD cavity. The NMR titration data supported a 1:1 stoichiometry. The assignments of the proton NMR resonances in the absence of the host are in accordance with previous reports^31^. However, the chemical shifts observed for the phenyl and imazilyl protons (H-3, 5, 6, 10, 11, and 13) were downfield (Table S1 in the Supporting Information) by increasing the concentration of β-CD (up to 1.5 equivalents), which means that they are located outside of the β-CD portal (inset in Fig. 2B). However, the chemical shifts for the aliphatic protons (H-15, 16, and 17) were upfield (by 0.1 ppm) with the addition of β-CD because they are located within the β-CD cavity (Table S1 in the Supporting Information). The complexation-induced shifts in the aliphatic region are consistent with preferential inclusion of the aliphatic protons over the aromatic protons (inset in Fig. 2B). The observation in the current study were also rationalized based on this literature precedent on the interaction of IMZ with β-CD host^31^.

The shifts in the proton resonances of β-CD upon completion to IMZ have further established the engulfing of guest molecules. For example, H-3 and H-5 protons, which are directed towards the interior of the host cavity showed a significant upfield (Table S2 in the Supporting Information), whereas H-1, H-2 and H-4 protons, which are positioned towards the exterior of the cavity exhibited marginal chemical shifts (Table S2 in the Supporting Information). It transpires that a total inclusion can be inferred for IMZ/β-CD system in which the shift in H-5 proton (Δδ) are larger than that of H-3 proton (−0.09 versus −0.03) in agreement with the conclusion made by others on different host-guest complexes of CDs^32^. It is worth to mention that similar NMR pattern (Figure S2 in the Supporting Information) were observed upon the inclusion of CAD inside β-CD. The high variation of chemical shifts in Figure S2 in the Supporting Information for the proton located inside the cavity (H-3 and H-5) coupled with the lack of variations in those resides at the torus of β-CD (H-1, H-2 and H-4) provided a clear evidence for the embedding process of guest molecules. The later experiment was necessary to show the potential of CAD for competitive displacement of IMZ from the host cavity.

**Figure 3:**
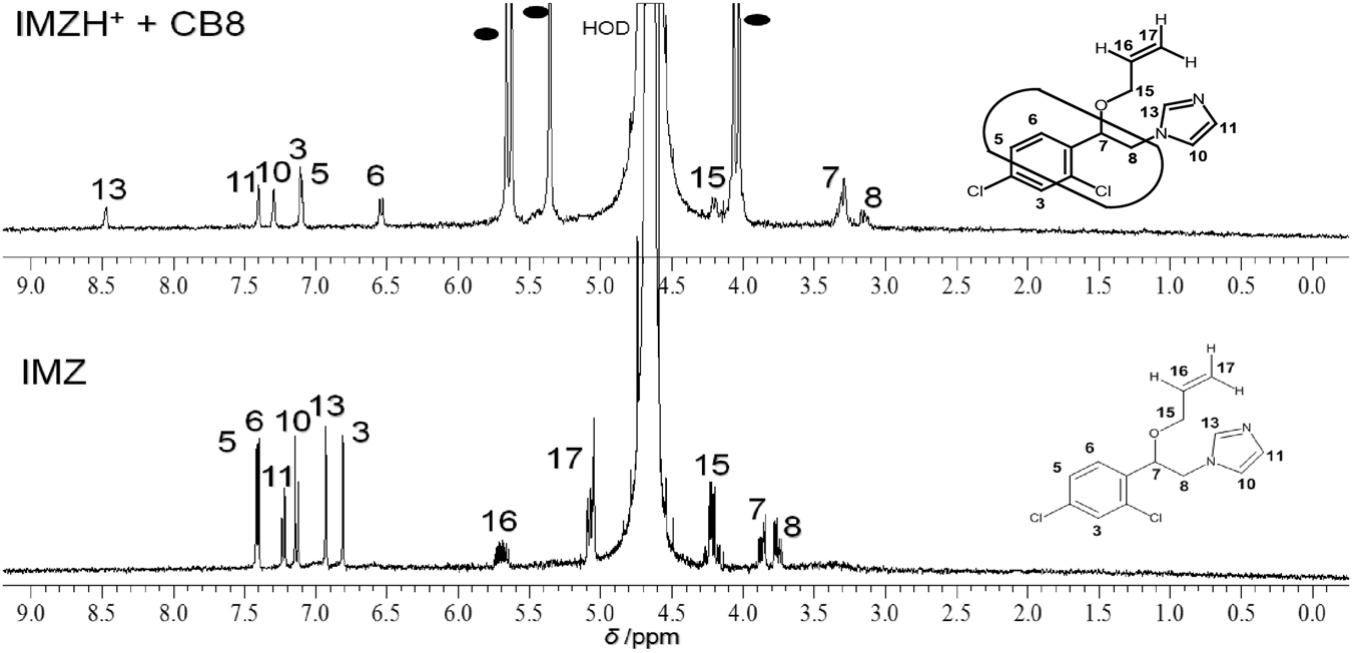
^1^H-NMR spectra of free IMZ (bottom spectrum) and CB8-complexed IMZ (2.0 equiv.) in D_2_O at pD 8.0 (400 MHz. HOD = solvent peak and filled squares = CB8 peaks.

NMR spectra of neutral IMZ in the absence and presence of CB8 in D_2_O supported an inclusion from aromatic site, opposite to the observed complexation-induced shifts inside β-CD (Fig. 3). While, the chemical shifts observed for (H-13, 10, 11, and 3) were downfield by increasing the concentration of CB8 (up to 2.0 equivalents), which means that they are located outside of the CB8 portal, the chemical shifts for the phenyl protons (H-5 and 6) were upfield (by 0.3–0.9 ppm) with the addition of CB8 because they are located within the CB8 cavity. Aliphatic protons (H-7 and 8) were also upfiled (by 0.4–0.5 ppm) with the addition of CB8, which means they are also embedded within the host cavity. While protons 16 and 17 were hidden under the host peaks, proton 15 appears to exhibit no shift, indicating its positioning at the portal. Moreover, H-13 protons appears to be distinctly shifted to a higher ppm over other protons of imazilyl ring (1.6 ppm vs. 0.1–0.2 ppm), due to stabilization of protonated form inside CB8 cavity, which is rationalized based on a literature precedent on the interaction of benzimidazoles with CBs hosts^10^. The later conclusion is also supported by similar complexation-induced shifts inside CB8 of the protonated IMZ observed at pD 2 (top spectrum in Figure S3A and B in the Supporting Information), when compared to observed results of CB8-bound IMZ at pD 8 (top spectrum in Fig. 3). Data in Figures S4 and S5 in the Supporting Information support a significant interactions of the protonated IMZ (*K* ~ 2 × 10^6^ M^−1^) and 1:1 binding stoichiometry from the Job’s plot. The shifts of the resonances to lower frequencies observed for the protons mentioned above are because of the shielding effect of the hydrophobic cavity produced inside β-CD and CB8, while shifting the protons to higher frequencies is because of the deshielding effects by the proximity of the negatively charged carbonyl groups of CB8, as an example.

### Validation of stimuli-responsive abilities by NMR measurements

**Figure 4:**
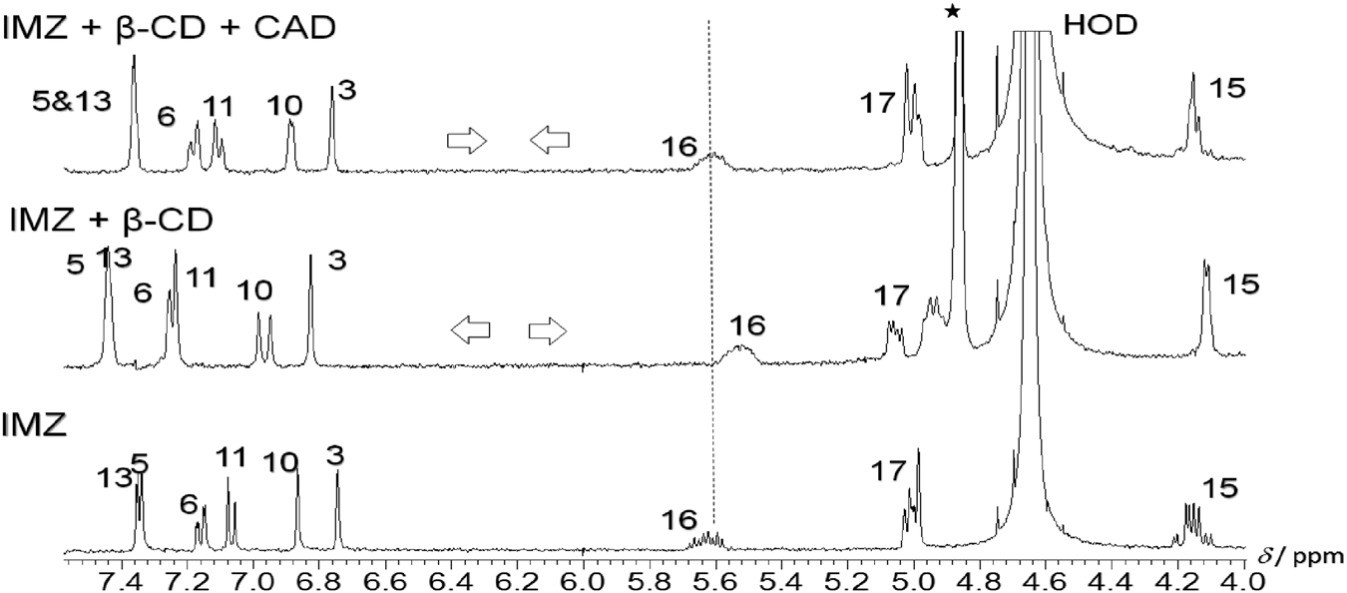
^1^H-NMR spectra of free IMZ (bottom spectrum) and β-CD-complexed IMZ (1.5 equiv.) without (bottom spectrum); and with CAD (5 equiv.; top spectrum) in D_2_O at pD 8.0 (400 MHz. HOD = Solvent Peak and *β-CD peaks.

The stimuli-responsive abilities were confirmed by observing efficient restoring of NMR spectrum of IMZ upon the addition of CAD to β-CD-bound IMZ complex signifying the replacement of drug from the host cavity. For example, while aromatic protons 13,5,6,11,10, and 3 were shifted to higher ppm, the aliphatic protons 16,17, and 15 were shifted to high field upon complexation to β-CD, but then their peak positions were restored when CAD was added (Fig. 4). Protons 7 and 8, however, displayed opposite trend by shifting initially downfield, before shifting back towards their original value (data not shown). Furthermore, the peak profile for each proton has undergone a significant change, such as that of proton 15, which appeared as doublet of doublet, before merging with the addition of host. That peak splitting was, noticeably, restored in the presence of CAD. Protons 13, 6 and 11 behaved similarly, whereas the singlet-doublet switching of proton 10 appeared in an opposite order. Noteworthy, the re-merging of peaks pertinent to the two H-17 protons upon addition of CAD (Fig. 4).

**Figure 5:**
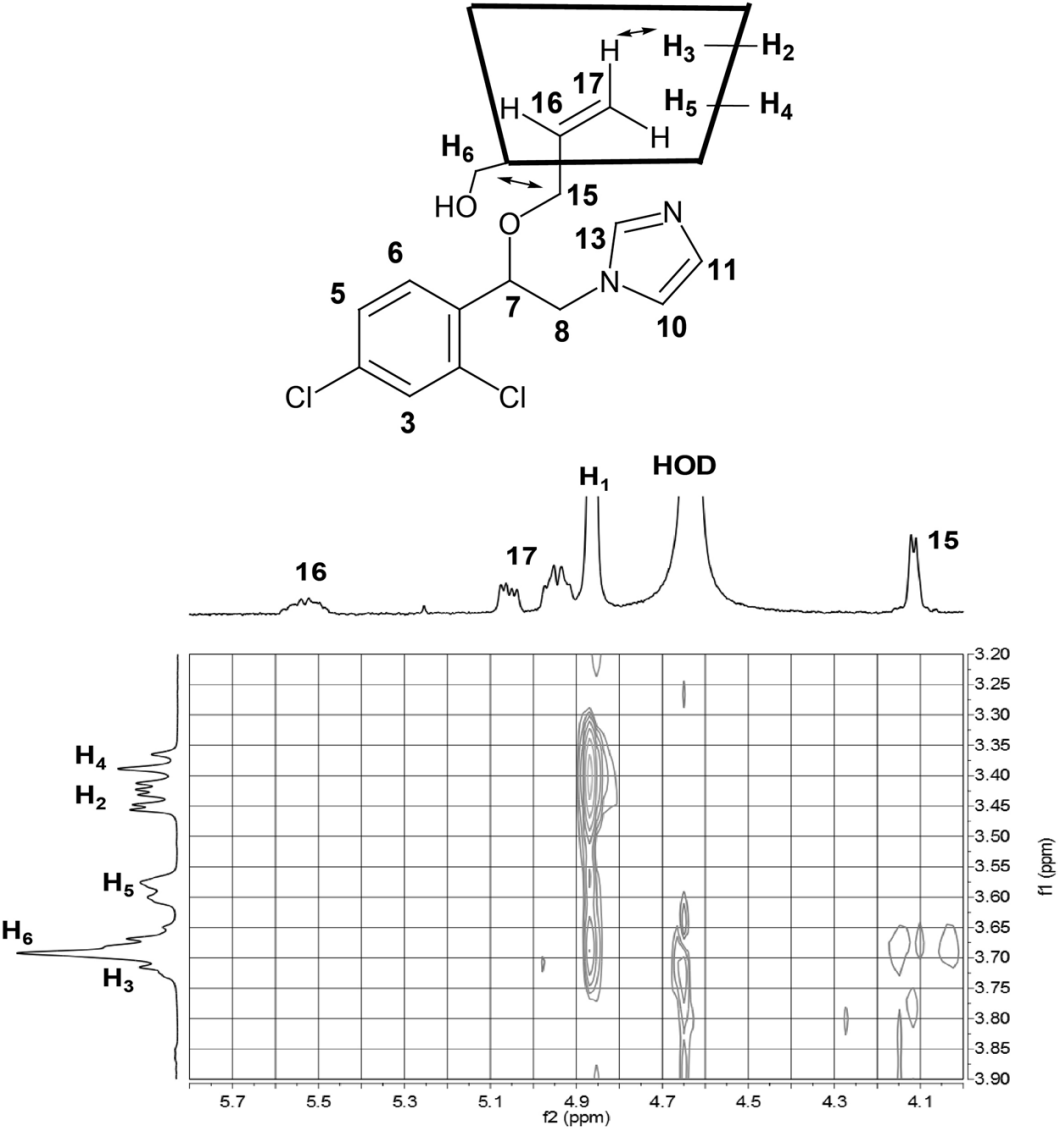
Partial contour plot of the NOESY spectrum of IMZ/β-CD complex (1.5 equiv of the host) at pD 8.0. The *inset* shows the structure of the complex as concluded from the observed interactions in the spectrum.

To further proof the establishment of a stimuli-responsive system for IMZ/β-CD with CAD, we turned our attention to 2D-NMR characterization using NOESY plots. The partial contour plot and full NOESY spectrum of the IMZ/β-CD complex are reported in Figs 5 and S6 in the Supporting Information, respectively. The 2D spectrum shows intermolecular cross-peaks between H3 proton of β-CD at 3.71 ppm (Table S2 in the Supporting Information) and the H17 proton of IMZ at 4.98 ppm (Table S1 in the Supporting Information), demonstrating the inclusion of this part in the hydrophobic interior cavity of β-CD. Furthermore, the spectrum indicates the existence of cross-peaks between H15 proton of IMZ at 4.10 ppm and H6,6’ at 3.66 ppm of β-CD, indicating that the guest penetrates into the host cavity from the primary OH group side as shown in the inset of Fig. 5. The NOESY spectrum of the β-CD/IMZ/CAD complex is shown in Figure S7 in the Supporting Information. The 2D spectrum reveals the disappearance of the intermolecular interaction between protons of β-CD and those of IMZ, ensuring no sequestration of the guest into the host upon the addition of stimuli.

### Cadaverine-driven control of the antifungal activity

**Figure 6:**
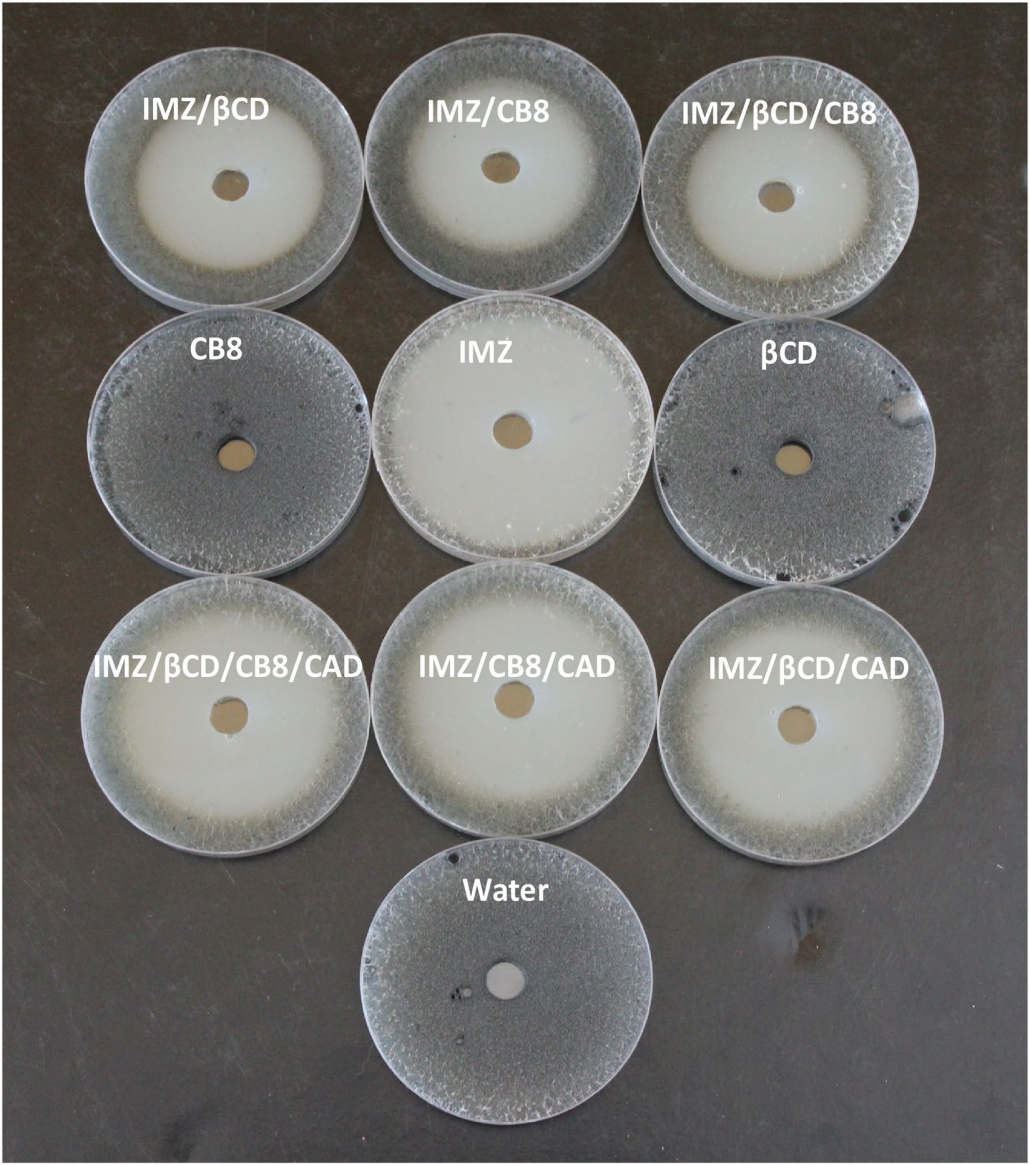
Effect of different tested compounds (codes were explained in Fig. 1) on the mycelial growth of *Thielaviopsis punctulata*. Wells were cut in the centers of the fresh PDA plates seeded with *T. punctulata* using a sterilized 11-mm cork-borer. Aliquots (0.5 ml) of the filter-sterilized chemicals and water (control) were pipetted into the wells using a sterilized syringe. The plates were incubated at 28 °C in dark and the diameters of inhibition zones were measured in mm after 4 days.

The ability to respond to CAD was validated against economically important plant pathogenic fungi, when the antifungal activity was turned down and up upon the addition of macrocycles and CAD, respectively. The antifungal activities of IMZ and complexes were tested against seven different economically important plant pathogenic fungi that gave variable results (Table 1, Fig. 6 and Figures S8–S13 in the Supporting Information). However, the trend is similar against all fungi tested in that encapsulation inside CB8 and β-CD (combined together or in separate) first reduced IMZ antifungal activity, then subsequent restoring of activity was achieved, on demand, upon triggering the system with CAD (Table 1). CAD alone showed no antifungal activities against all the fungi tested using the cup plate method (Figure S14 in the Supporting Information).

**Table 1:** Effect of different tested compounds (codes were explained in Fig. 1) on mycelial growth of seven economically important plant pathogenic fungi using the cup plate method. Diameter of inhibition zones were measured in mm after 4 days of incubation at 28 °C.

Tested compound	*Thielaviopsis punctulata*	*Ulocladium atrum*	*Cladosporium cladosporioides*	*Mauginiella scaettae*	*Fusarium oxysporum*	*Alternaria solani*	*Helminthosporium solani*
IMZ/β-CD	51.82 ± 0.65 *c*	28.61 ± 0.41*c*	32.26 ± 0.37 *c*	41.18 ± 0.42 *c*	43.87 ± 0.84 *c*	31.78 ± 0.51 *c*	44.42 ± 0.97 *c*
IMZ/CB8	52.36 ± 0.23 *c*	28.46 ± 0.32 *c*	31.20 ± 0.81 *c*	42.05 ± 0.40 *c*	44.77 ± 0.65 *c*	32.61 ± 0.34 *c*	43.34 ± 0.57 *c*
IMZ/β-CD/CB8	52.28 ± 0.42 *c*	29.41 ± 0.56 *c*	32.00 ± 0.61 *c*	41.63 ± 0.35 *c*	44.30 ± 0.64 *c*	33.14 ± 0.88 *c*	42.74 ± 0.71 *c*
IMZ	79.51 ± 0.49 *a*	72.91 ± 0.87 *a*	46.70 ± 0.53 *a*	62.79 ± 0.68 *a*	68.50 ± 0.50 *a*	62.62 ± 1.04 *a*	67.46 ± 0.41 *a*
IMZ/β-CD/CAD	62.91 ± 0.49 *b*	41.85 ± 0.44 *b*	40.90 ± 0.50 *b*	51.33 ± 0.14 *b*	54.21 ± 0.40 *b*	45.37 ± 0.71 *b*	54.32 ± 0.69 *b*
IMZ/CB8/CAD	62.15 ± 0.36 *b*	42.42 ± 0.37 *b*	40.00 ± 0.31 *b*	51.77 ± 0.47 *b*	55.64 ± 0.91 *b*	44.64 ± 0.57 *b*	53.36 ± 0.88 *b*
IMZ/β-CD/CB8/CAD	62.00 ± 0.50 *b*	42.74 ± 0.53 *b*	40.61 ± 0.62 *b*	52.03 ± 0.59 *b*	55.33 ± 0.69 *b*	46.01 ± 0.43 *b*	52.69 ± 0.91 *b*
Control (water, CAD)	0.00 ± 0.00 *d*	0.00 ± 0.00 *d*	0.00 ± 0.00 *d*	0.00 ± 0.00 *d*	0.00 ± 0.00 *d*	0.00 ± 0.00 *d*	0.00 ± 0.00 *d*
CB8	0.00 ± 0.00 *d*	0.00 ± 0.00 *d*	0.00 ± 0.00 *d*	0.00 ± 0.00 *d*	0.00 ± 0.00 *d*	0.00 ± 0.00 *d*	0.00 ± 0.00 *d*
β-CD	0.00 ± 0.00 *d*	0.00 ± 0.00 *d*	0.00 ± 0.00 *d*	0.00 ± 0.00 *d*	0.00 ± 0.00 *d*	0.00 ± 0.00 *d*	0.00 ± 0.00 *d*

Each value is a mean of five replicates ± standard error. Values with the same letter within a column are not significantly (P > 0.05) different according to TukeyTest. Concentration of each ingredient was fixed at 100 micromolar in pure water at pH 8. Biological tests were performed after 1 week from preparation.

## Discussion

1-[2-(allyl-oxy)-2-(2,4-dichlorophenyl)ethyl]-1*H*-imidazole (named enilconazole or imazalil) was selected for two reasons. On the one hand the drug was effective against fungi that are of major concerns in the agricultural sector of the United Arab Emirates. For example, *Thielaviopsis punctulata* is causing a serious disease called black scorch disease of date palm and *Mauginiella scaettae* Cavara is the causal agent of inflorescence rot disease of date palm. On the other hand, we pursued a biocide whose fungicidal efficiency can be significant at low dose (such as 100 micromolars), due to the limited solubility of our selected macrocycles. The experiments were all conducted at pH 8 at which neutral IMZ persists (see Figure S1 in the Supporting Information). Thus IMZ was a good model to achieve the target in the present study, as outlined in the introduction. In one attempt (data not shown), changing the protonable form of IMZ gave no change in the observed trend of data in agreement with previous results on other drugs^33^.

In the present study, the selection of β-CD was based on a precedent study by Schirra *et al*.^31^, in which after 4 days the IMZ/β-CD complex was more effective against *Penicillium digitatum* and *P. italicum* than the free IMZ, whilst the freshly prepared β-CD/IMZ was as effective as IMZ^31^. The authors^31^ proposed the drug was gradually released from the complex, thereby increasing its bioavailability. Our results with seven different economically important plant pathogenic fungi (Table 1) showed that complexation to either β-CD or CB8 or both has negative influence on IMZ activity, even when measurements were performed after 1 week. These differences in the trend between our results and the results obtained by Schirra *et al*.^31^, who did their experiment only against *Penicillium digitatum* and *P. italicum* may be explained by the common occurrence of inter- and intra- specific variations in fungicide resistance among strains of fungal taxa. Such variations have been widely reported in both pathogenic and non-pathogenic fungi as suggested previously by others^34^. In the present work, the complex was not isolated in solid state, but used upon mixing each gradient at 1:1 molar ratio in solution, without heating for 2 h. Interestingly, the trend in our results does not depend on the type of macrocycles or fungi tested, indicating a common mechanism of drug action that still needs further investigations. CB8 is known to interact with amine-containing guest through ion-dipole, which clearly explains the difference in its binding mode when compared to β-CD^7^. Having such specificity towards binding site did not influence IMZ bioavailability, which also helps unfolding the mechanism of IMZ biocidal activity.

**Figure 7:**
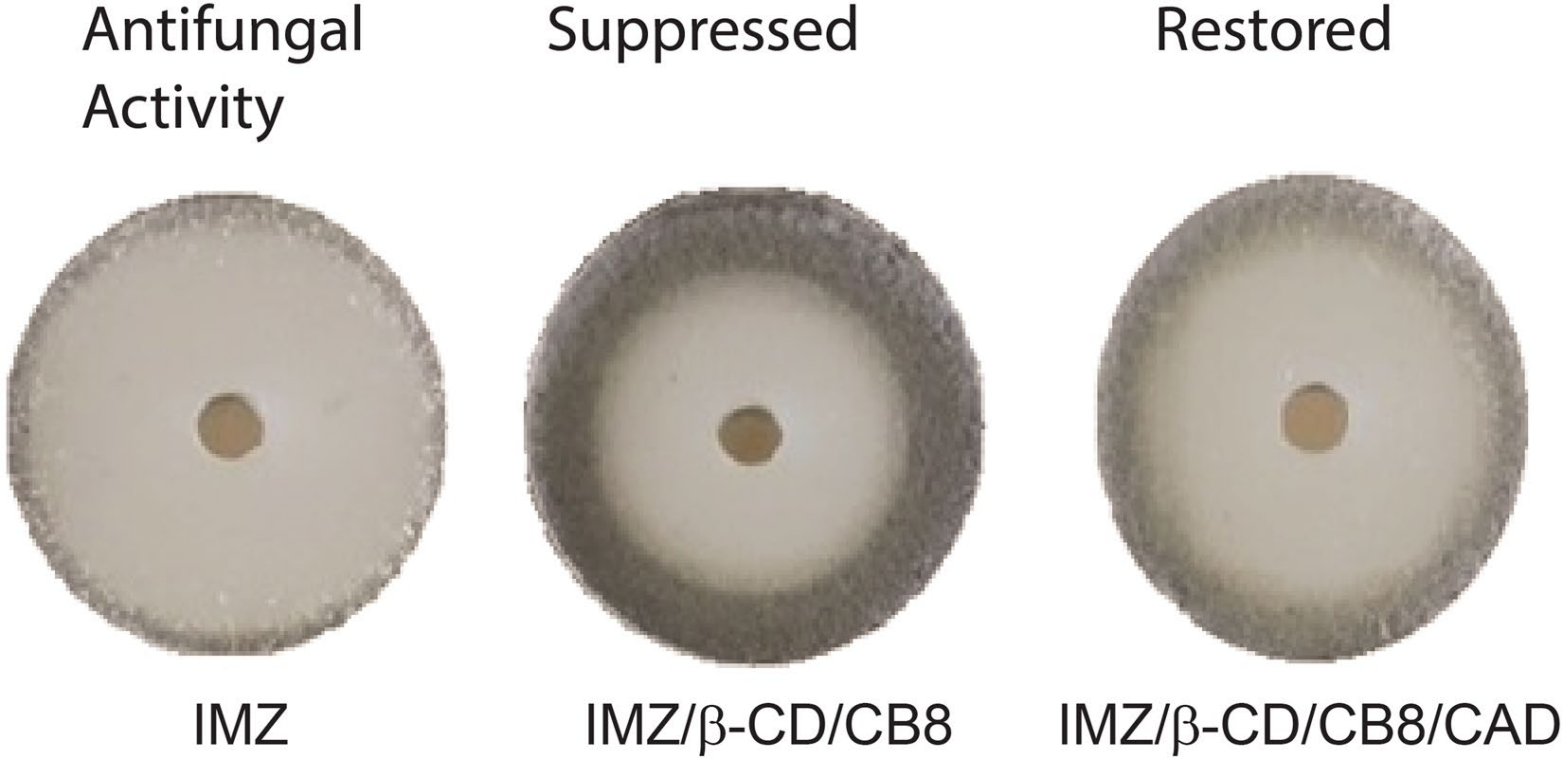
Schematic illustration that summarizes how antifungal activity of imazalil (IMZ) against *Thielaviopsis punctulata* (DCM 102798) the causal agent of black scorch disease of date palm can be controlled on demand in response to cadaverine (CAD) stimulus utilizing cucurbit[8]uril (CB8) and β-cyclodextrins (β-CD) macrocycles.

Even though similar demonstrations have already been reported in mammalian cell lines^25–28^ and despites few other researchers even reported stimuli responsive assemblies *in vivo*^29^, the results in this current article distinctly reveal a clear harmony between chemical and biological validations on the ability to manipulate on demand biological activity in living fungi utilizing supramolecular approach for the first time (Fig. 7). CAD is known to have a high binding affinity towards CB and was already used in other precedent reports to breakdown host-guest assembles^7^. It is expected that 100% restoring cannot be achieved in principle, as supramolecular association is inherently reversible being non-covalent and chances for formation of an exclusion complex that could have some residual effects cannot be overlooked. The results, however, reveal thermodynamic control that is induced by CAD, whose preferential binding towards macrocycles over IMZ plays a major role.

In summary, the present study gives experimental data that demonstrate the ability to chemically manipulate antifungal activity in living fungal cells utilizing macrocycles. In the present example, IMZ, cyclodextrins, cucurbiutrils and CAD are used as a proof-of-concept (Fig. 7). There have been several reports on the use of macrocyles to increase or decrease antifungal activity^35^, but only the present study gives a clear evidence on how host-guest interactions endow the system with ability to respond to external chemical stimuli. Having the ability to modulate biological activities in a controlled manner should enforce our understanding of drug mechanism of action and find an economical value on the industrial use of fungi for chemical production.

## Materials and Methods

### Chemicals

Imazalil (IMZ), cucurbit[8]uril (CB8), and β-cyclodextrin (β-CD) were purchased from Sigma-Aldrich Chemie GmbH, Taufkirchen, Germany (purity 99%). D_2_O, DCl and NaOD were also purchased from Sigma-Aldrich. Millipore water had conductivity less than 0.05 μS. The pH values of the solutions were adjusted (±0.2 units) by adding adequate amounts of HCl (DCl) or NaOH (NaOD) and were recorded using a pH meter (WTW 330i equipped with a WTW SenTix Mic glass electrode).

### Optical and NMR measurements

The UV-Visible absorption spectra were measured on Cary-300 instrument (Agilent). ^1^H-NMR spectra were performed on a Varian 400 MHz spectrometer in D_2_O and referenced in ppm with respect to a TMS standard. For the NMR titration experiment, the pD of a certain volume of D_2_O was first adjusted to either 2 or 8 in which a stock solution of IMZ was prepared to give a final concentration of ~1.5 mM. A calculated weight of β-CD or CB8 was added to the same solution of IMZ to prepare the stock solution of the complex (about 2.5 mM). The solutions with the final concentration of CB8 were prepared by gradually adding increment volumes of the complex’s stock solution to 1 ml of the free IMZ directly in the NMR tubes. The NMR spectra were measured for each solution. For the job’s plot experiment, two stock solutions were prepared by measuring the calculated weights, which results in a given ratio of concentrations with the total concentration fixed at 3 mM for [CB8] + [IMZ].

### Binding affinity measurements

In the titration experiment, the total concentrations of the IMZ were kept constant and that of the host was gradually increased. The NMR peak positions were plotted as a function of host’s total concentrations. The intermolecular interaction between CB8 (as an example) and IMZ may be quantified by the affinity constant referred to as the association equilibrium (*K*):

$${\rm{IMZ}}+{\rm{CB8}}\rightleftharpoons \mathrm{IMZ}/\mathrm{CB8}$$

1 $$K=\frac{[IMZ/CB8]}{[IMZ][CB8]\,}$$

2 $${{\rm{C}}}_{{\rm{IMZ}}}=[{\rm{IMZ}}]+[\mathrm{IMZ}/\mathrm{CB8}]$$

3 $${{\rm{C}}}_{{\rm{C}}{\rm{B}}8}=[{\rm{C}}{\rm{B}}8]+[{\rm{I}}{\rm{M}}{\rm{Z}}/{\rm{C}}{\rm{B}}8],$$

where C_IMZ_ and C_CB8_ mean the total concentrations of IMZ and CB8, respectively. It can be written that:

4 $${\rm{Y}}\,({\rm{Reading}}\,{\rm{at}}\,{\rm{the}}\,{\rm{ppm}})={\rm{constant}}\,{\rm{1}}\ast [{\rm{IMZ}}]+{\rm{constant}}\,{\rm{2}}\ast [\mathrm{IMZ}/\mathrm{CB8}]$$

Using Eqs (1–4), we obtain

5 $${\rm{\Delta }}{{\rm{Y}}}^{ppm}=\frac{{\rm{\Delta }}({\rm{constant}}){C}_{CB8}}{\frac{2}{K{C}_{IMZ}-1-K{C}_{CB8}+\sqrt{{(1-K{C}_{IMZ}+K{C}_{CB8})}^{2}+4K{C}_{IMZ}}}+1},$$

where ΔY = NMR changes at a given ppm; Δ(constant) = the difference between constants 1 and 2, and *K = *binding constant. The binding constants (*K*) were then evaluated by using the nonlinear formula of Eq. (5). Constant 2 was left as a floating parameter in the analysis by Levenberg-Marquardt algorithm, which was provided by SigmaPlot’s software (version 6.1; SPCC, Inc., Chicago, Illinois, USA).

### Fungal growth

The seven plant pathogenic fungi used in the present study were cultured on potato dextrose agar (PDA; Lab M Limited, Heywood, Lancashire, UK) plates, pH 6.0; supplemented with ampicillin (Sigma-Aldrich Chemie GmbH, Taufkirchen, Germany) used at a rate of 25 mg l^−1^ of growing medium to inhibit the bacterial contaminants. For every particular fungus, mycelium-containing agar was sub-cultured to fresh PDA plates every 10 days and incubated at 28 °C.

The fungi used were as follows: *Thielaviopsis punctulata* (Hennebert) Paulin, Harrington & McNew (DSM 102798) the causal agent of black scorch disease of date palm; *Ulocladium atrum* Preuss (DSM 63068) the causal agent of leaf spots of orchard grass; *Cladosporium cladosporioides* (Fresenius) de Vries the causal agent of rot disease of grapes; *Mauginiella scaettae* Cavara, the causal agent of inflorescence rot disease of date palm; *Fusarium oxysporum* Schlechtendahl: Fries (DSM 21731) the causal agent of wilt disease of sugar beet; *Alternaria solani* (Ellis et Martin) Sorauer (ATCC 58177) the causal agent of early blight disease of tomato; and *Helminthosporium solani* Durieu et Montagne (ATCC 204193) the causal agent of silver scurf of potato.

### Detection of the antifungal activity of the chemicals used using the cup plate method

We aimed to screen the tested compounds (IMZ, IMZ/β-CD, IMZ/CB8, IMZ/β-CD/CB8, IMZ/β-CD/CAD, IMZ/CB8/CAD, and IMZ/β-CD/CB8/CAD) for their potential to produce diffusible antifungal metabolites active against the above mentioned fungi using the cup plate method as described by Bacharach and Cuthbertson^36^. This method determined the extent of inhibition of mycelial growth. Various methods have been used to monitor fungal growth and inhibition due to antifungal activity of chemical compounds. Although optical density (OD) has sometimes been used to determine the inhibitory activity of antifungal compounds, the disadvantage of using OD especially for filamentous fungi, however, is that actively growing hyphae are not evenly distributed and this might give uncertainties in the quantitative estimation of fungal growth^37^. In addition, sporulation might give unexpectedly high OD values and thereby overestimation of mycelial growth^37^. OD is therefore, at best, suitable for early detection of mold growth or for growth versus no growth observations^37^. OD, however is more appropriate for the enumeration of unicellular organisms (e.g. bacteria and yeasts) suspended uniformly in liquid media^37^. Alternative methods for fungal growth quantification includes measure of fungal biomass, and measuring the diameter of the colony or the inhibitory zone around molds^37–40^. Accordingly, in our current study, we used the cup plate method^36^ which is based on determination of diameter of inhibition zone. In literature^37–40^, the cup plate method^36^ and the measurement of the diameter of inhibition zone has routinely been used to quantify levels of inhibition by antifungal metabolites^37–40^.

The effect of CAD, β-CD and CB8 were also tested for their potential to produce diffusible antifungal metabolites active against the above mentioned fungi using the same cup plate method^36^ described below. Concentration of each ingredient was fixed at 100 micromolar in pure water at pH 8. The tested compounds were filtered through sterile Millipore membranes of pore size 0.22 µm (Millipore Corporation, MA, USA) and collected in sterile tubes which were stored in the refrigerator at 4 °C until use. Inocula for the preparation of the PDA-fungal seeded plates were prepared by cultivating every fungus individually on PDA slants at 28 °C until abundant sporulation occurred. The slant surfaces were then flooded with 50 mM phosphate buffer (pH 6.8), and the spores as well as some mycelial fragments were dislodged by scraping the surface growth with a sterilized scalpel. The spore and the mycelial fragments were then homogenized in an Omni-mixer (OCI Instruments, Omni Corporation International, Waterbury, CT, USA) at 4000 rpm for 20 min. The resultant suspensions were then diluted and added to sterile cooled PDA prior to the pouring of the plates. A suspension of approximately 10^8^ CFU ml^−1^ was used as inoculum. Wells (11 mm in diameter) were cut in the centres of the fresh PDA plates seeded with every fungus using a sterilized 11-mm cork-borer. Aliquots (0.5 ml) of every filter-sterilized tested compound were pipetted into the wells using a sterilized syringe. The plates were incubated at 28 °C in dark for 4 days; and the diameters of zones of inhibition were measured in mm. Filter sterilized distilled water was similarly pipetted into the wells in the PDA plates seeded with every fungus to serve as controls. Five replicates for every tested compound for every fungus were used.

### Statistical analysis

All data were subjected to a one-way analysis of variance (ANOVA) to test the effects of the different tested compounds on the mycelial growth of the seven tested fungi. Significant differences among treatment means were compared using Tukey test at P = 0.05. SAS Software version 9 was used for all statistical analysis performed^41^.

## Electronic supplementary material

Supplementary File
