# Supplementary material for: Host-guest complexes of imazalil with cucurbit[8]uril and β-cyclodextrin and their effect on plant pathogenic fungi
Source: Sci Rep. 2018 Feb 12;8:2839. doi: 10.1038/s41598-018-21156-9 (PMC5809605; doi:10.1038/s41598-018-21156-9)
Supplement: Supplementary file 1 — Supplementary File [file 41598_2018_21156_MOESM1_ESM.pdf]

SUPPORTING INFORMATION

Host-guest complexes of imazalil with cucurbit[8]uril and β-cyclodextrin and their effect on plant pathogenic fungi

Naji Al-Dubaili,[a] Khaled El-Tarabily,[b] Na'il Saleh,*[a]

[a]*Chemistry Department, College of Science, United Arab Emirates University, P.O.Box 15551, Al-Ain, United Arab Emirates*

[b]*Biology Department, College of Science, United Arab Emirates University, P.O.Box 15551, Al-Ain, United Arab Emirates*

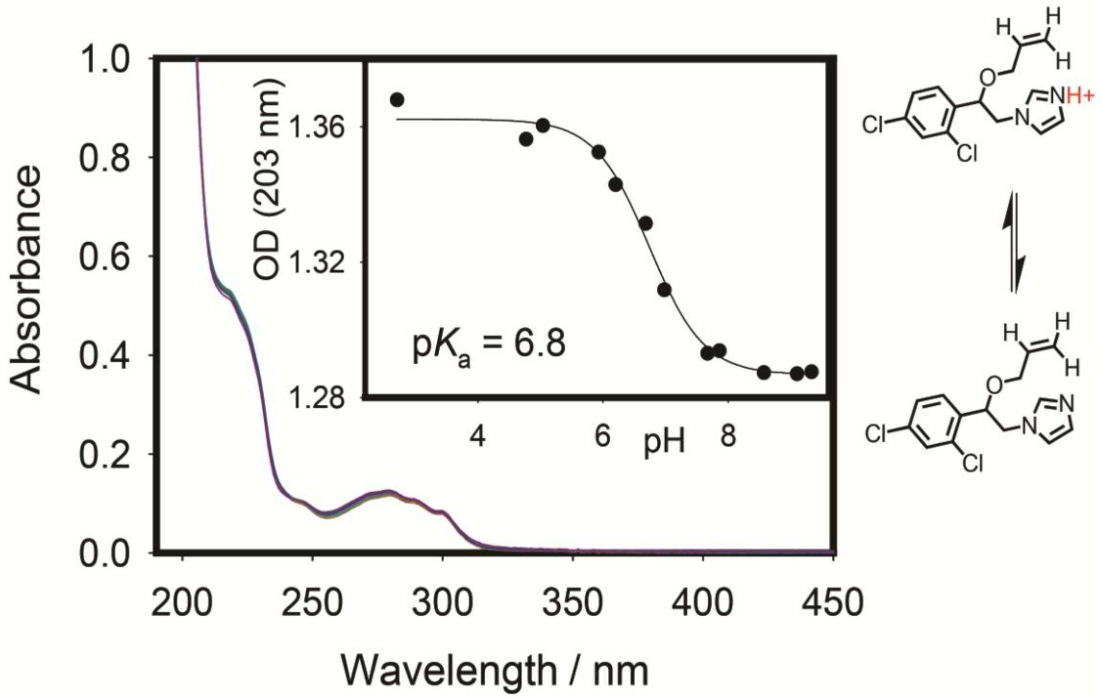

Figure S1. UV–Visible absorption spectra of IMZ in aqueous solutions at different pH values: 2–10. The insets show the experimental fit to a sigmoidal function, which gives pK_a values of 6.41 ± 0.06 for the corresponding spectra in A. The same pK_a values were obtained if other wavelength was selected (200, 220, and 233 nm). The left panel shows the chemical structures of each forms result from protonation-deprotonation equilibrium of the nitrogen at position-12. The absorption spectral profiles of IMZ in aqueous solutions at different pH values in Figure S1 were explained as changing the pH of the media affects both the shape and position of absorption band maxima. These changes are corresponding to the protonation processes because of the intermolecular interactions of the protonable nitrogen at position-12 with water molecules.

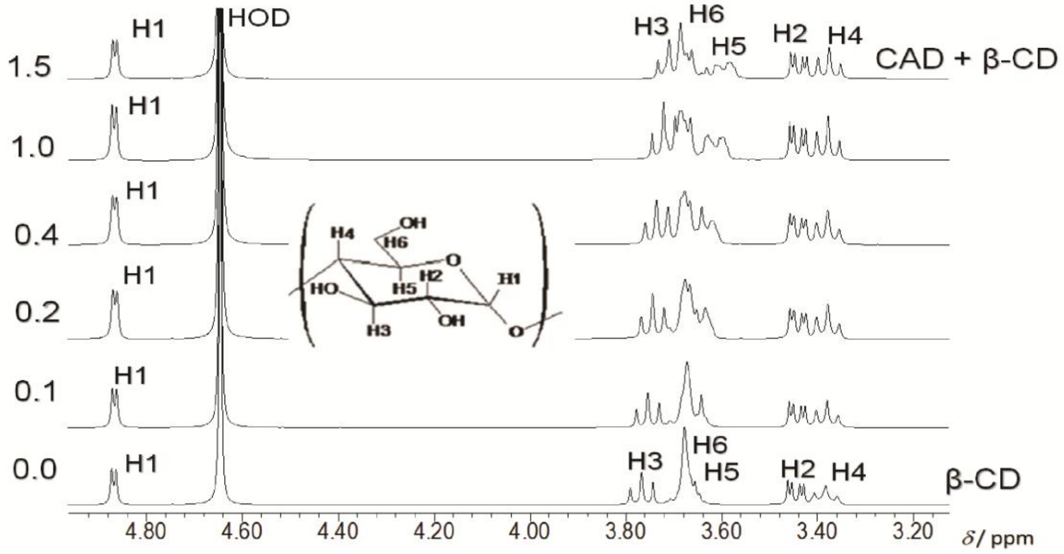

Figure S2. ^{1}H-NMR titration of β-CD with CAD (0 – 1.5 equiv.) in D_2O at pD 8.0 (400 MHz).

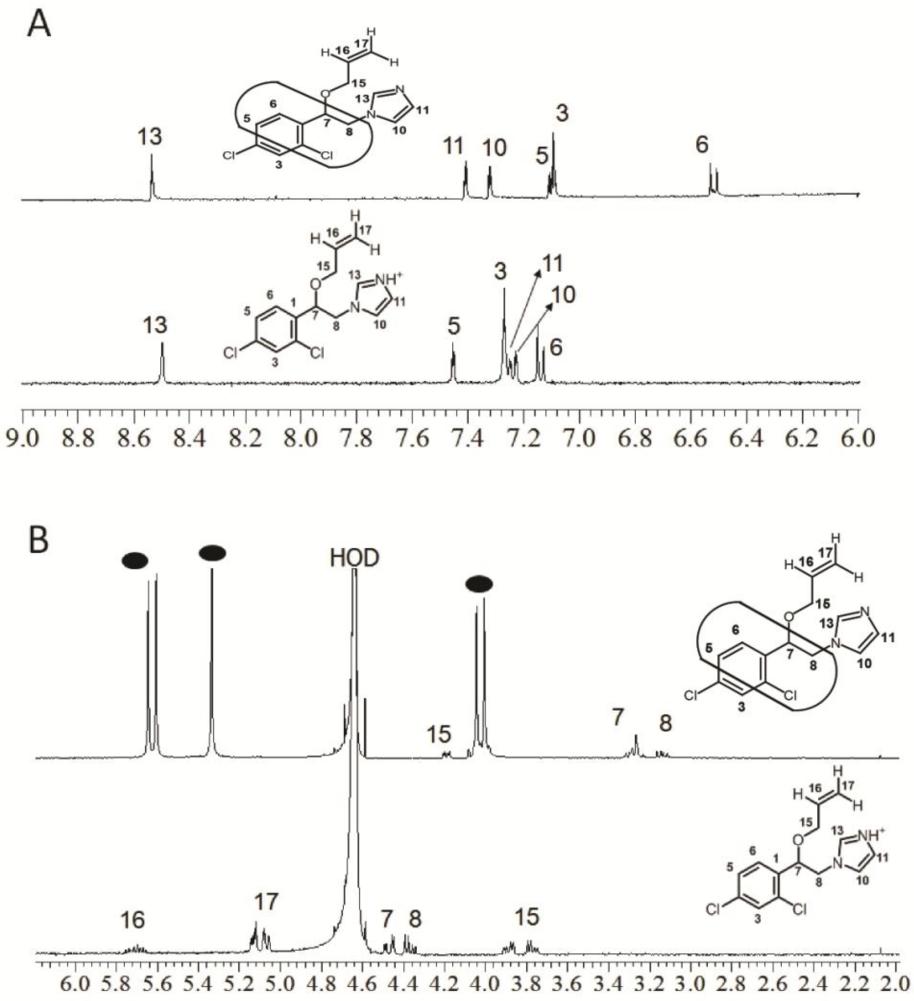

Figure S3. ^{1}H-NMR spectra of free IMZ (bottom spectrum) and CB8-complexed IMZ (2.0 equiv.) in D_2O at pD 2.0 (400 MHz. HOD = solvent peak and filled squares = CB8 peaks. A) Aromatic region, and B) aliphatic region.

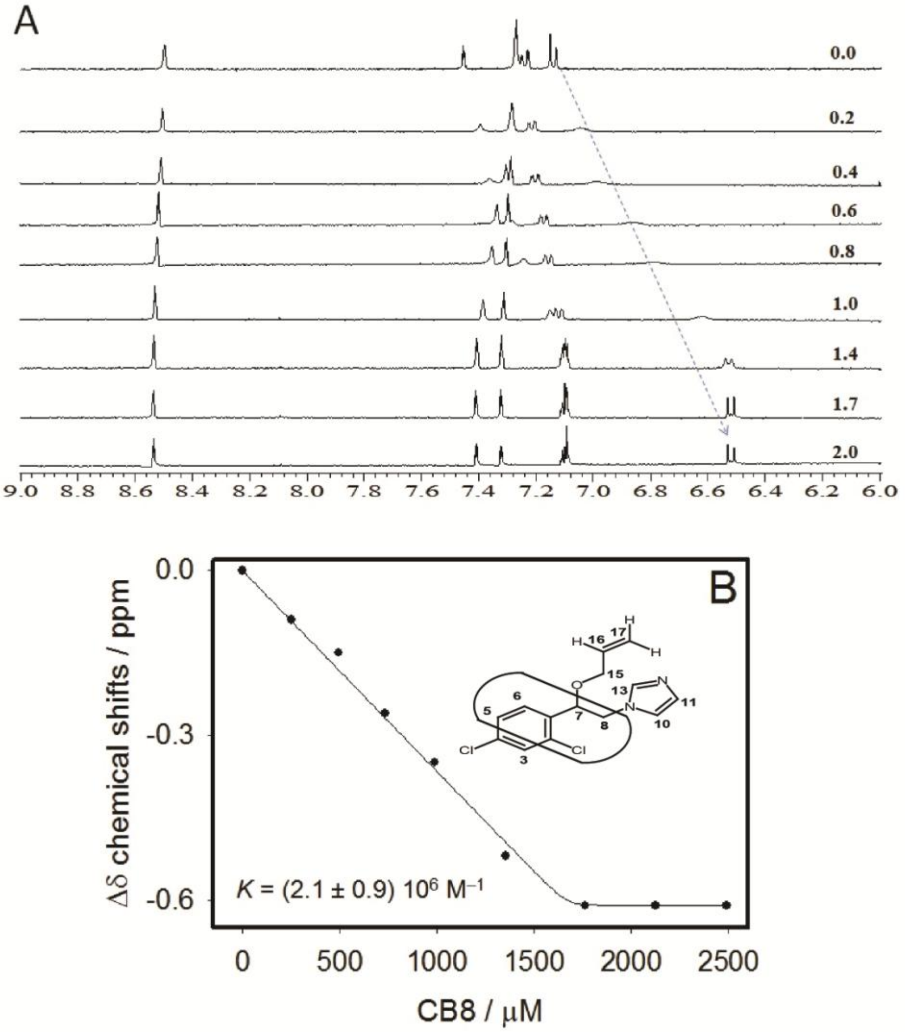

Figure S4. ^{1}H-NMR titration of IMZ with CB8 (0 – 2.0 equiv.) in D_2O at pD 2.0 (400 MHz): (A) Spectral changes; (B) Nonlinear fitting plot of the chemical shift (ppm) at ~6.5 ppm versus concentration of CB8 in M. K was evaluated as $(2.1 \pm 0.9) \times 10^{6}$ M^{-1}. The inset in B shows a schematic representation of the resulted complex.

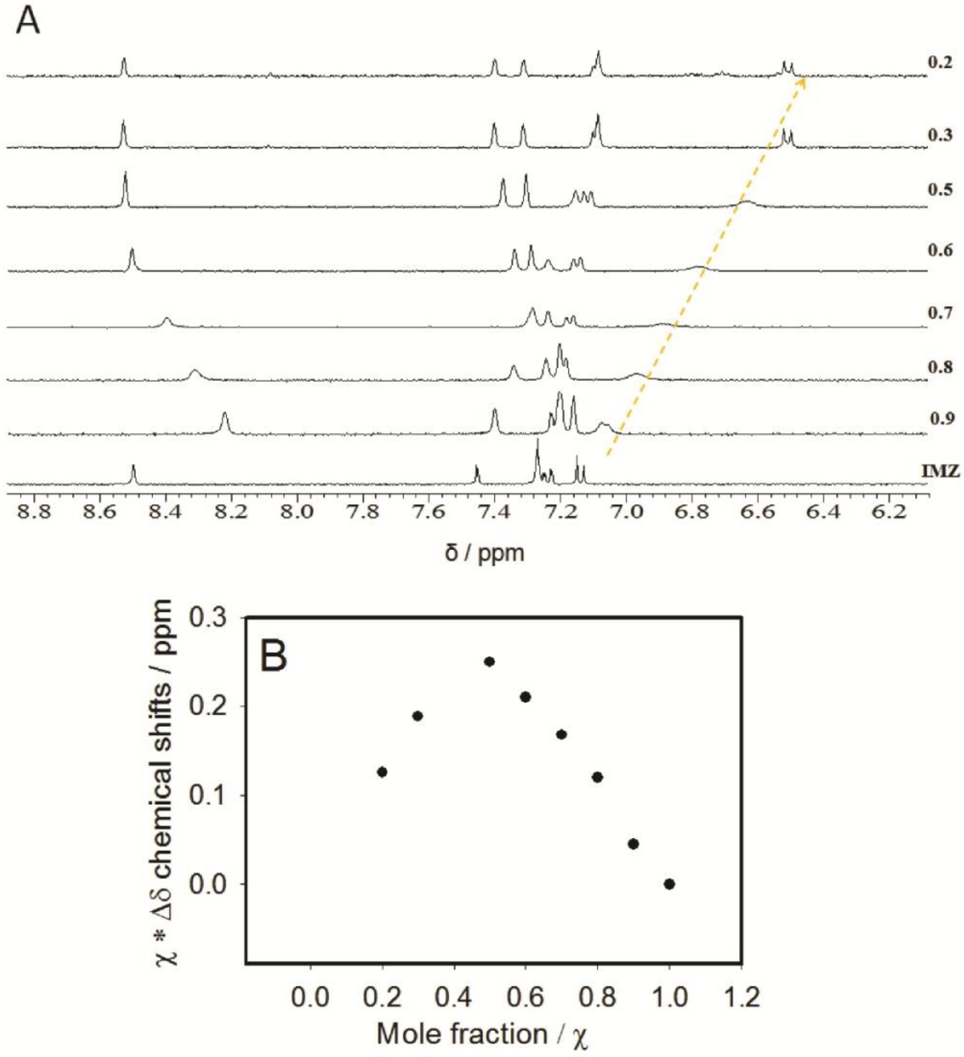

Figure S5. ^{1}H-NMR spectra at different ratios of IMZ and CB8 ([IMZ] + [CB8] = 3 mM) at pD 2.0. The peak at ~7.2 ppm in the bottom spectra was monitored; (A) spectral changes; (B) Job's plot constructed from the data in part (A), which indicates 1:1 stoichiometry. Note that Δδ was taken as the difference between the observed chemical shifts in the presence of varying mole fractions of CB7 and the intrinsic shift of the drug in the absence of CB8.

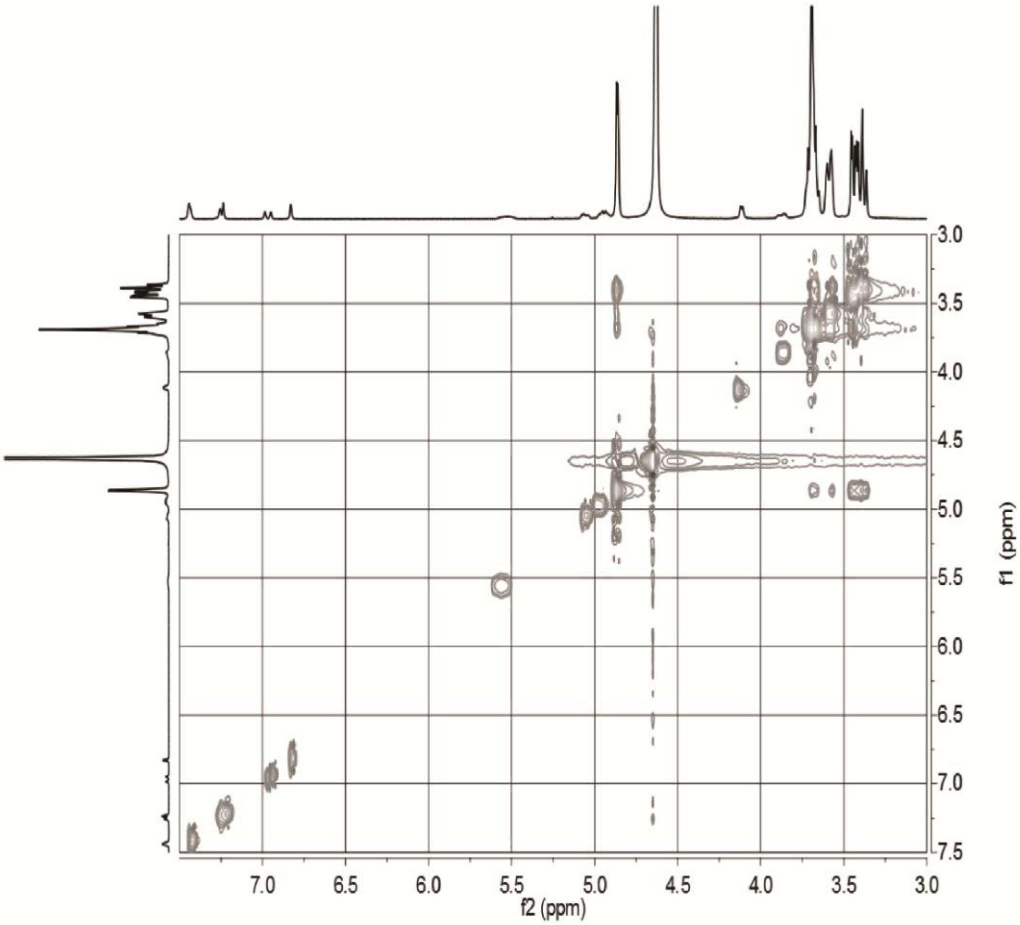

Figure S6. NOESY spectrum of IMZ/β-CD complex.

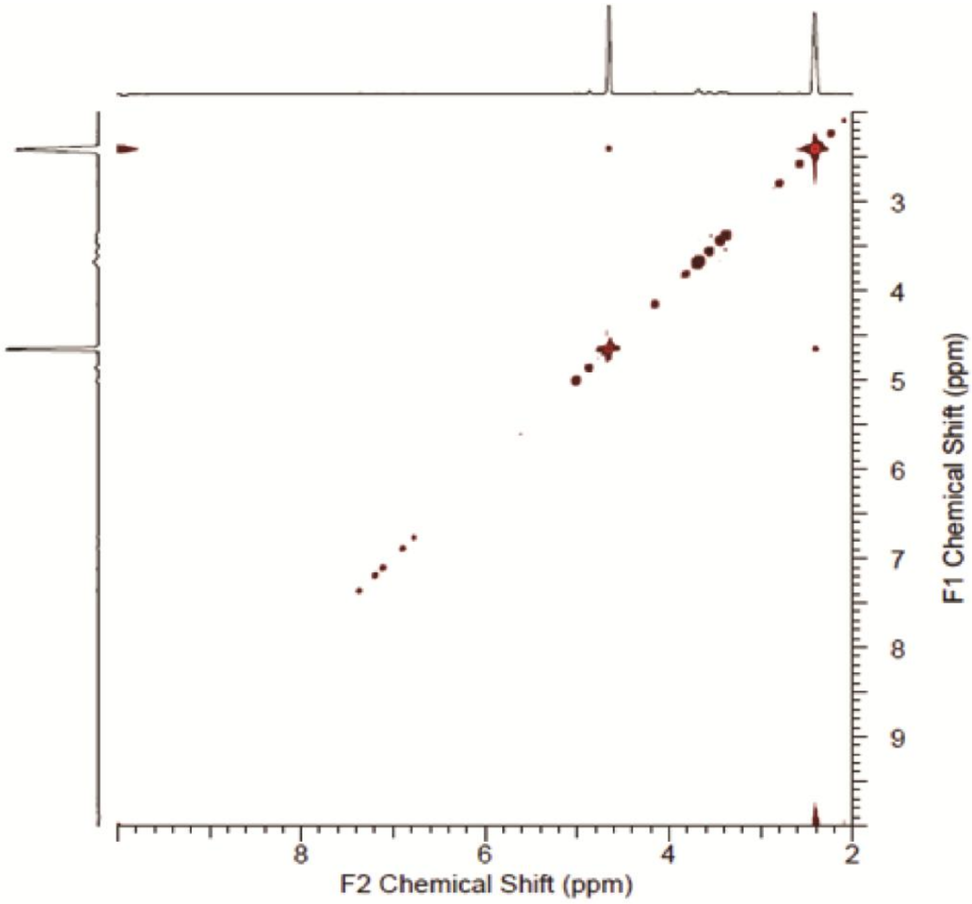

Figure S7. NOESY spectrum of IMZ/β-CD/CAD complex (0-8 ppm)

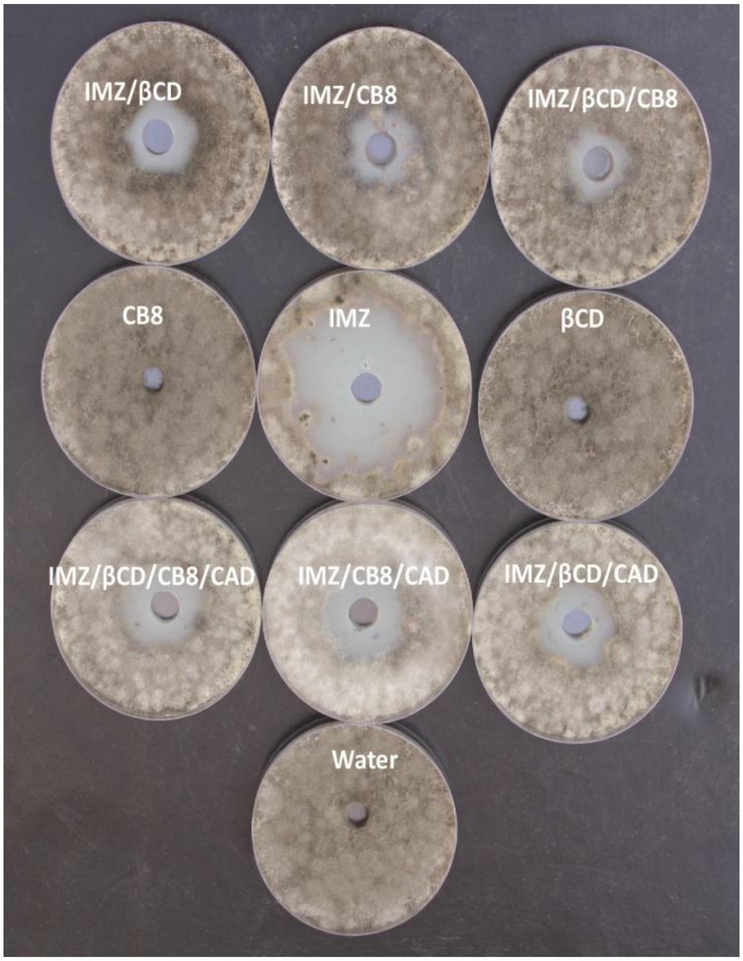

Figure S8. Effect of different tested compounds (codes were explained in Figure 1) on the mycelial growth of *Ulocladium atrum*. Wells were cut in the centers of the fresh PDA plates seeded with *U. atrum* using a sterilized 11-mm cork-borer. Aliquots (0.5 ml) of the filter-sterilized chemicals and water (control) were pipetted into the wells using a sterilized syringe. The plates were incubated at 28°C in dark and the dimeters of inhibition zones were measured in mm after 4 days.

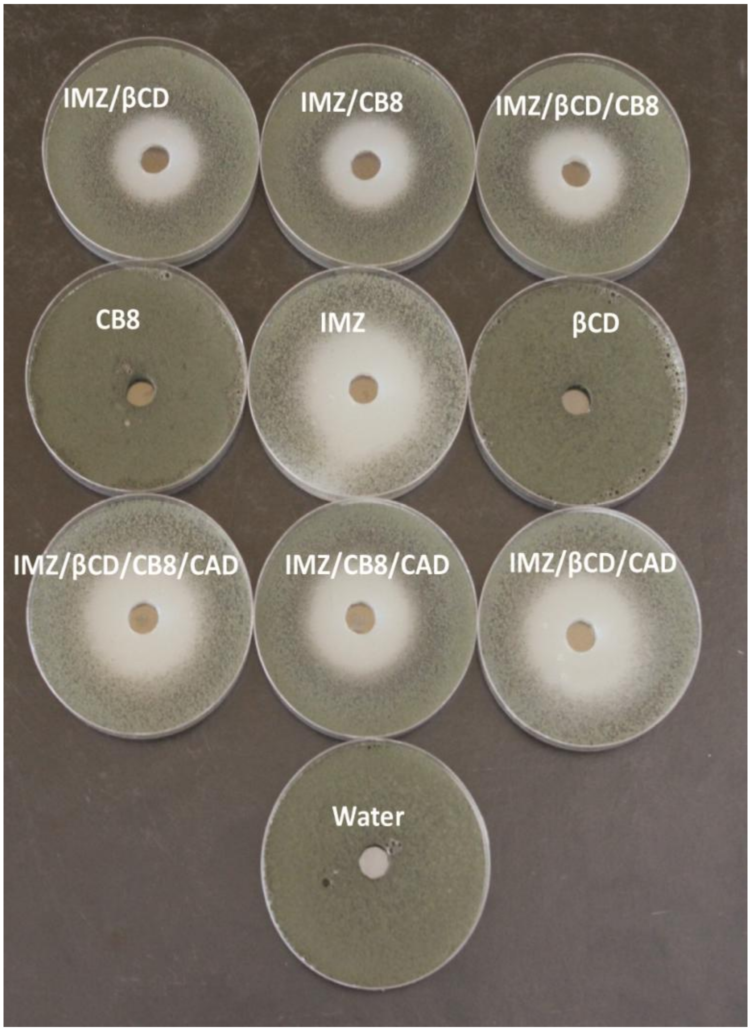

Figure S9. Effect of different tested compounds (codes were explained in Figure 1) on the mycelial growth of *Cladosporium cladosporioides*. Wells were cut in the centers of the fresh PDA plates seeded with *C. cladosporioides* using a sterilized 11-mm cork-borer. Aliquots (0.5 ml) of the filter-sterilized chemicals and water (control) were pipetted into the wells using a sterilized syringe. The plates were incubated at 28°C in dark and the dimeters of inhibition zones were measured in mm after 4 days.

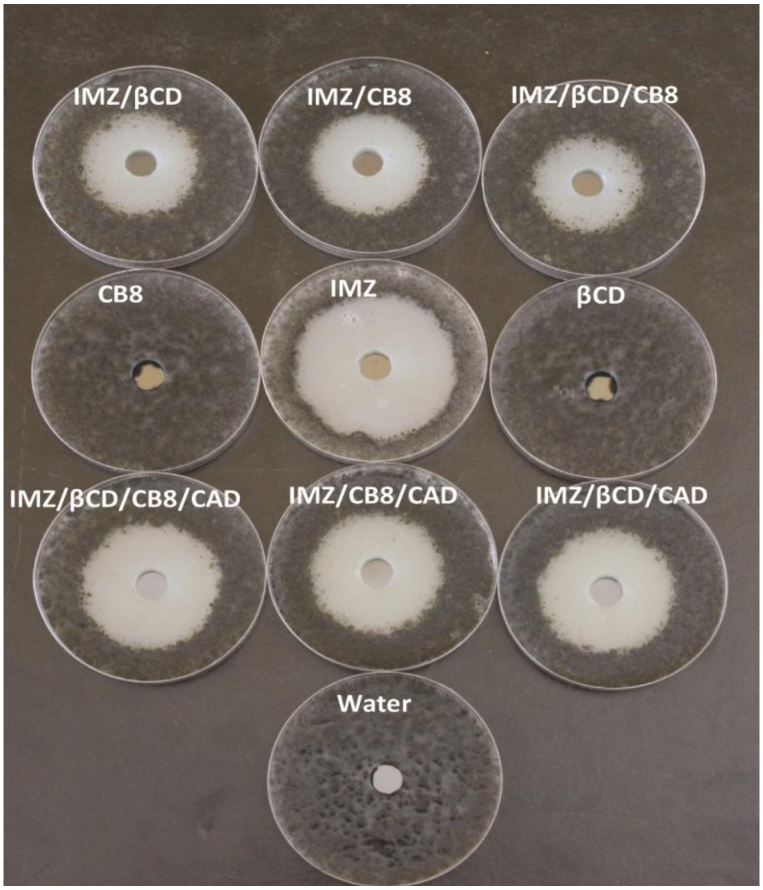

Figure S10. Effect of different tested compounds (codes were explained in Figure 1) on the mycelial growth of *Mauginiella scaettae*. Wells were cut in the centers of the fresh PDA plates seeded with *M. scaettae* using a sterilized 11-mm cork-borer. Aliquots (0.5 ml) of the filter-sterilized chemicals and water (control) were pipetted into the wells using a sterilized syringe. The plates were incubated at 28°C in dark and the dimeters of inhibition zones were measured in mm after 4 days.

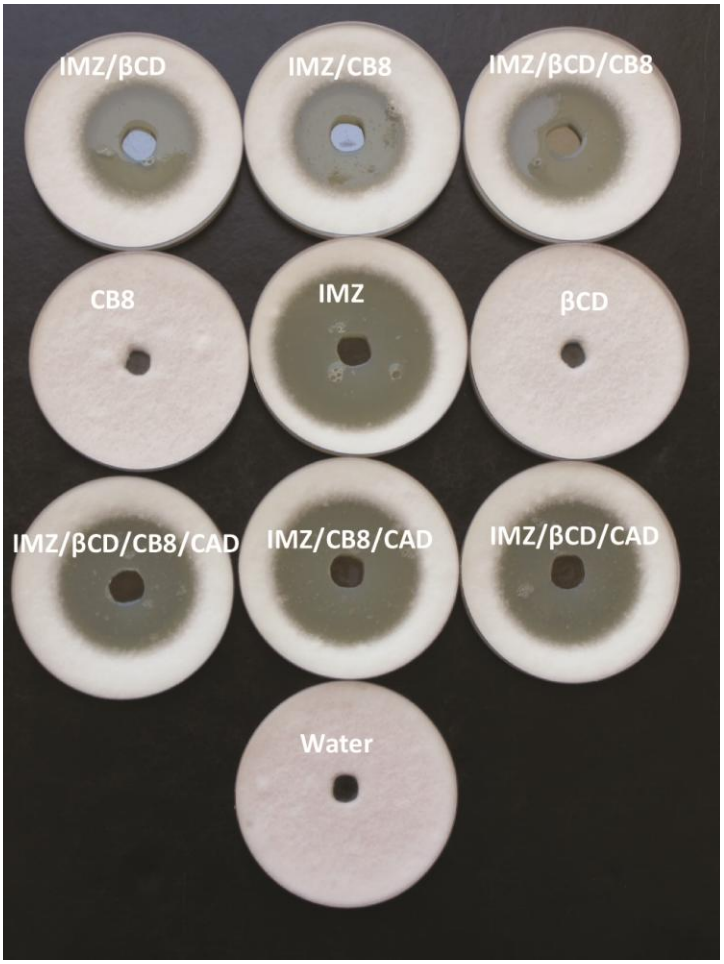

Figure S11. Effect of different tested compounds (codes were explained in Figure 1) on the mycelial growth of *Fusarium oxysporum*. Wells were cut in the centers of the fresh PDA plates seeded with *F. oxysporum* using a sterilized 11-mm cork-borer. Aliquots (0.5 ml) of the filter-sterilized chemicals and water (control) were pipetted into the wells using a sterilized syringe. The plates were incubated at 28°C in dark and the dimeters of inhibition zones were measured in mm after 4 days.

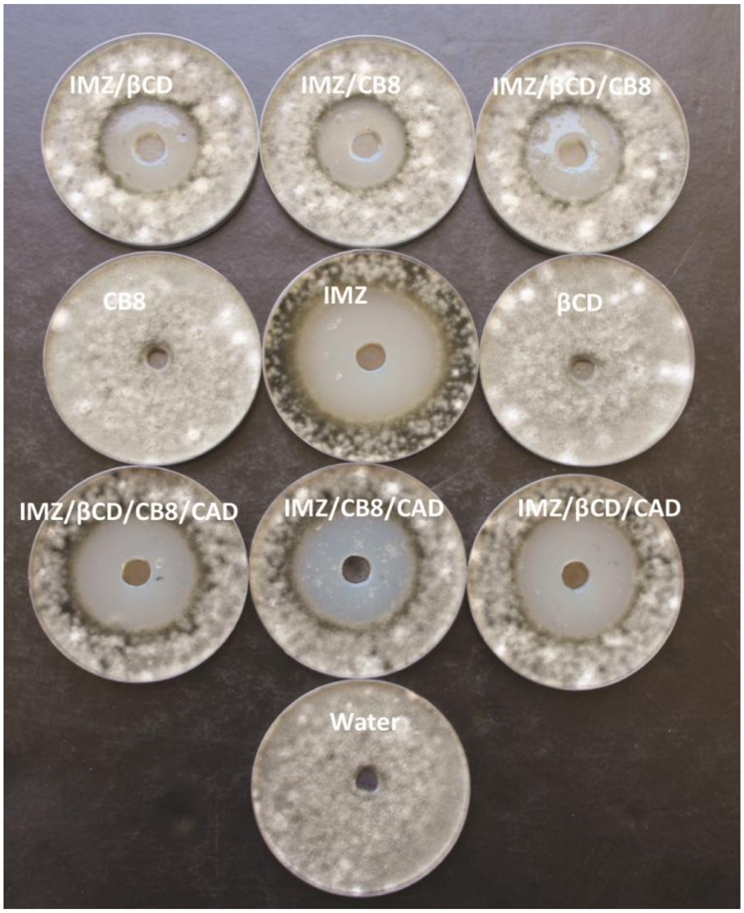

Figure S12. Effect of different tested compounds (codes were explained in Figure 1) on the mycelial growth of *Alternaria solani*. Wells were cut in the centers of the fresh PDA plates seeded with *A. solani* using a sterilized 11-mm cork-borer. Aliquots (0.5 ml) of the filter-sterilized chemicals and water (control) were pipetted into the wells using a sterilized syringe. The plates were incubated at 28°C in dark and the dimeters of inhibition zones were measured in mm after 4 days.

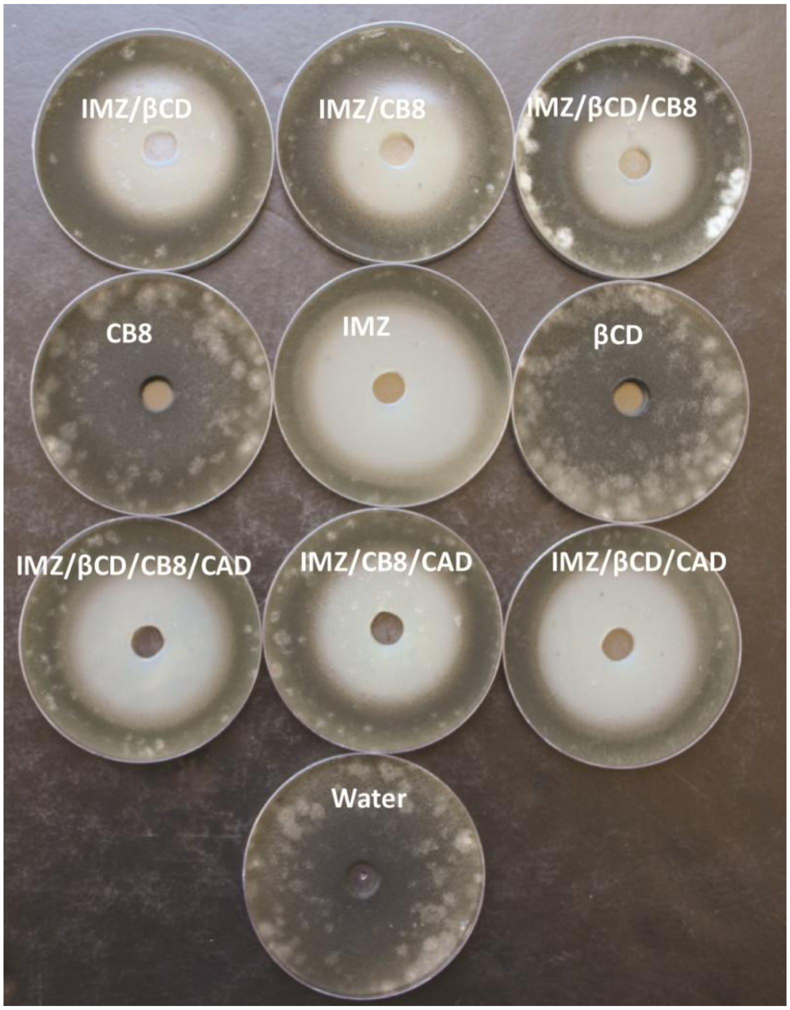

Figure S13. Effect of different tested compounds (codes were explained in Figure 1) on the mycelial growth of *Helminthosporium solani*. Wells were cut in the centers of the fresh PDA plates seeded with *H. solani* using a sterilized 11-mm cork-borer. Aliquots (0.5 ml) of the filter-sterilized chemicals and water (control) were pipetted into the wells using a sterilized syringe. The plates were incubated at 28°C in dark and the dimeters of inhibition zones were measured in mm after 4 days.

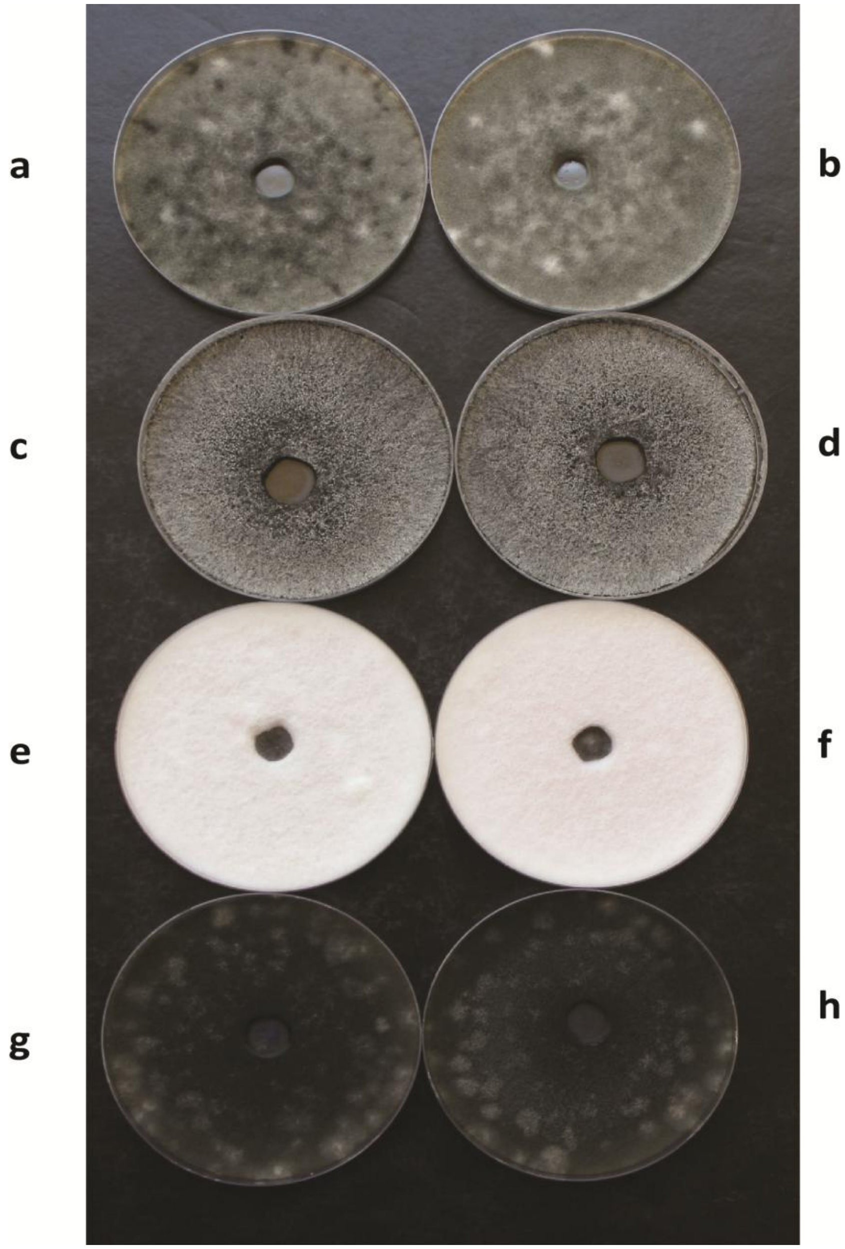

Figure S14. The effect of cadaverine on the growth of different fungi: (b) *Alternaria solani*, (d) *Thielaviopsis punctulata*, (f) *Fusarium oxysporum* and (h) *Helminthosporium solani*. Wells were cut in the centers of the fresh PDA plates seeded with every fungus using a sterilized 11-mm cork-borer. Aliquots (0.5 ml) of the filter-sterilized cadaverine were pipetted into the wells (b, d, f, and h) or filter-sterilized water (a, c, e, and g) as controls using a sterilized syringe. The plates were incubated at 28°C in dark and the dimeters of inhibition zones were measured in mm after 4 days.

Table S1: Proton chemical shifts in ppm (Δδ) of free and β-CD-complexed IMZ (d: doublet, t: triplet, q: quadruplet, m: multiplet)

Proton	δ Free	*δ* Complex	Δδ
H-3	6.74	6.81	+0.07
H-10	6.86	6.95	+0.09
H-11	7.06	7.23	+0.17
H-6	7.15	7.25	+0.10
H-5	7.34	7.45	+0.11
H-13	7.35	7.45	+0.10
H-16	5.65	5.55	−0.10
H-17	5.00	4.98	−0.02
H-15	4.15	4.10	−0.05
H-7	3.80	3.85	+ 0.05
H-8	3.67	3.81	+ 0.14

Table S2: Proton chemical shifts in ppm (Δδ) of free and IMZ-complexed β-CD (d: doublet, t: triplet, q: quadruplet, m: multiplet)

Proton	δ Free	*δ* Complex	Δδ
H-1 (1H,d)	4.86	4.85	-0.01
H-2 (1H,d)	3.43	3.43	0
H-3 (1H,t)	3.74	3.71	-0.03
H-4 (IH,t)	3.36	3.36	0
H-5 (1H,d)	3.65	3.56	-0.09
H-6,6′ (2H,t)	3.66	3.66	0
